# Identification of plasma protein markers common to patients with malignant tumour and Abnormal Savda in Uighur medicine: a prospective clinical study

**DOI:** 10.1186/s12906-015-0526-6

**Published:** 2015-02-05

**Authors:** Halmurat Upur, Yin Chen, Mayila Kamilijiang, Wanli Deng, Xierzhatijiang Sulaiman, Renaguli Aizezi, Xiao Wu, Wuniqiemu Tulake, Abulizi Abudula

**Affiliations:** Key Laboratory of High-Incident Diseases in Uyghur Ethnic Population supported by the Chinese Ministry of Education, Xinjiang Medical University, 393 Xinyi Rd., Urumqi, 830011 P R China; Oncology Center, Affiliated General Hospital of Chinese Medicine, Xinjiang Medical University, 393 Xinyi Rd., Urumqi, 830011 P R China; Department of Biochemistry and Molecular Biology, Xinjiang Medical University, 393 Xinyi Rd., Urumqi, 830011 P R China; School of Uighur Medicine, Xinjiang Medical University, 393 Xinyi Rd., Urumqi, 830011 P R China

**Keywords:** Uighur medicine, Abnormal Savda, Malignant tumour, Plasma proteomics

## Abstract

**Background:**

Traditional Uighur medicine shares an origin with Greco-Arab medicine. It describes the health of a human body as the dynamic homeostasis of four normal Hilits (humours), known as Kan, Phlegm, Safra, and Savda. An abnormal change in one Hilit may cause imbalance among the Hilits, leading to the development of a syndrome. Abnormal Savda is a major syndrome of complex diseases that are associated with common biological changes during disease development. Here, we studied the protein expression profile common to tumour patients with Abnormal Savda to elucidate the biological basis of this syndrome and identify potential biomarkers associated with Abnormal Savda.

**Methods:**

Patients with malignant tumours were classified by the diagnosis of Uighur medicine into two groups: Abnormal Savda type tumour (ASt) and non-Abnormal Savda type tumour (nASt), which includes other syndromes. The profile of proteins that were differentially expressed in ASt compared with nASt and normal controls (NC) was analysed by iTRAQ proteomics and evaluated by bioinformatics using MetaCore™ software and an online database. The expression of candidate proteins was verified in all plasma samples by enzyme-linked immunosorbent assay (ELISA).

**Results:**

We identified 31 plasma proteins that were differentially expressed in ASt compared with nASt, of which only 10 showed quantitatively different expression between ASt and NC. Bioinformatics analysis indicated that most of these proteins are known biomarkers for neoplasms of the stomach, breast, and lung. ELISA detection showed significant upregulation of plasma SAA1 and SPP24 and downregulation of PIGR and FASN in ASt compared with nASt and NC (p < 0.05).

**Conclusions:**

Abnormal Savda may be causally associated with changes in the whole regulation network of protein expression during carcinogenesis. The expression of potential biomarkers might be used to distinguish Abnormal Savda from other syndromes.

**Electronic supplementary material:**

The online version of this article (doi:10.1186/s12906-015-0526-6) contains supplementary material, which is available to authorized users.

## Background

In *Hippocratic Corpus* (c.410 to c.360 B.C.), the earliest texts of Greco-Arab medicine, Hippocrates used the words carcinos (crab) or carcinoma to describe a range of tumours and swellings caused by the excess of Black Bile, an opinion further developed by Claudius Galenus (c.200 A.D.) [1]. Traditional Uighur medicine shares an origin with Greco-Arab medicine, and describes the health of a human body as the dynamic homeostasis of four normal Hilits known as Kan, Phlegm, Safra, and Savda, which correspond to Blood, Phlegm, Yellow Bile, and Black bile in Greco-Arab medicine [2]. Imbalance of this homeostasis as a result of abnormal changes in any Hilit leads to the development of a corresponding syndrome coupled with different diseases [2,3]. Among these, Abnormal Savda (or abnormal Black Bile) is the predominant syndrome [3]. Long-term studies have established Abnormal Savda as the main syndrome of patients with various diseases, such as malignant tumours, type 2 diabetes mellitus, neurodegenerative diseases, depression, and cardiovascular diseases—conditions generally considered complex diseases in modern medicine (data not shown). Through a series of studies on Abnormal Savda from different aspects, including genetic susceptibility, metabolomics, the neuroendocrine–immunity network, oxidative stress, and pre-thrombotic state, we have demonstrated that Abnormal Savda is associated with common biological changes in different diseases that are manifested relatively consistently and holistically within a population [4-14]. The treatment of Abnormal Savda in patients with its unique prescription, a prescription composed of Abnormal Savda Munziq and Abnormal Savda Mushil, is well established in the clinical practice of Uighur Medicine [15]. Abnormal Savda Munziq may modulate the abnormal changes in the neuroendocrine–immunity network associated with Abnormal Savda, and prevent cells from carcinogenesis and oxidative damage [16-21]. Flavonoids isolated from Abnormal Savda Munziq may induce cell cycle arrest and cellular apoptosis of tumour cells [22-24]. These findings provide evidence for the biological basis of Savda (Black Bile), which is altered with the development of Abnormal Savda and restored by treatment with Abnormal Savda Munziq and Abnormal Savda Mushil.

Protein expression, the final stage of gene expression, changes dynamically in a spatiotemporal manner and varies with post-translational processing and chemical modifications. Recent advances in the study of malignant tumours by plasma-based proteomics have led to a better understanding of the regulatory network of plasma proteins that are altered with carcinogenesis and the identification of plasma protein markers for early diagnosis, prognosis, and treatment monitoring of malignant tumours of the stomach, lung, breast, colon, uterine cervix, and ovary [25-31]. These and other studies suggest that abnormal changes in plasma protein levels are associated with the overall pathologic state of tumour patients. This holistic concept of understanding tumour aetiology through systems biology is shared by the theories of traditional Uighur medicine.

In this study we investigated the profile of proteins that were differentially expressed in the plasma of patients with malignant tumour with Abnormal Savda compared with patients diagnosed with other syndromes in Uighur medicine using iTRAQ proteomics, bioinformatics, and quantitative verification, to elucidate the biological basis of Abnormal Savda and identify potential biomarkers. The findings of this study will contribute to the diagnosis of this syndrome and to monitoring the clinical treatment of malignant tumours using traditional Uighur medicines.

## Methods

### Diagnosis by Uighur medicine

The study design was approved and monitored by the Ethical Committee of the Hospital of Traditional Chinese Medicine affiliated to Xinjiang Medical University in Urumqi, China. All procedures of the study were followed in accordance with the Helsinki Declaration of 1975, as revised in 2000 (5). Informed consent was obtained from all donors, and the data were analysed anonymously throughout the study. Patients with breast, lung, and gastric cancer and healthy volunteers were enrolled in the study according to the diagnostic criteria of the World Health Organization and the Chinese Medical Association. The Abnormal Hilit (syndrome) type of each tumour patient, i.e., Abnormal Phlegm, Safra, Kan, or Savda, was defined by clinical diagnoses by two independent physicians of Uighur medicine according to established standard criteria that are described elsewhere in detail [15]. Subsequently, the patients were divided into either Abnormal Savda type tumour (ASt) or non-Abnormal Savda type tumour (nASt), which includes patients with other syndromes such as Abnormal Kan, Phlegm, or Safra. In addition, the classification was further defined for each patient by the assessment of symptom scores for Abnormal Savda, such as slow pulse, bleary eyes, dark-purple lips, blue tongue, cool skin temperature, dreams (or nightmares), night sweating, turbid urine, and dry stool.

### Clinical samples

Blood samples (2 mL) were obtained from each donor by venipuncture into evacuated blood collection tubes containing EDTA as an anticoagulant, and plasma samples separated by centrifugation were preserved at −80°C. Blood samples were collected from 136 tumour patients who met the inclusion criteria before any clinical therapy. Fifty age- and sex-matched healthy individuals were recruited as normal controls (NC) during routine physical check-ups in the hospital.

### Enrichment of low-abundance proteins

Plasma samples from 76 patients and 20 healthy individuals were subjected to proteomics analysis. The samples from patients with malignant tumours were divided into six subgroups representing three cancer types with further classification of patients as ASt or nASt as follows: 27 gastric cancer patients with 17 cases of ASt and 10 cases of nASt; 24 lung cancer patients with 14 cases of ASt and 10 cases of nASt; and 25 breast cancer patients with 13 cases of ASt and 12 cases of nASt. In addition, 20 normal controls were divided into two groups, with 10 cases in each. The samples from individuals in each group were mixed in equal volumes to form a pooled sample for further testing. Pooled samples were enriched for low-abundance proteins by depletion of medium- and high-abundance proteins using a prepacked 1-mL affinity LC column provided with the ProteoMiner™ Protein Enrichment Kit (Bio-Rad Laboratories, Richmond City, CA, USA) according to the manufacturer’s recommended procedure. After enrichment, the precipitated proteins were dissolved in 300 μL lysis buffer (6 M urea, 4% CHAPS, 1 mM PMSF, and 2 mM EDTA).

### Tryptic digestion and iTRAQ reagent labelling

After reduction (10 mM DTT, 100 mM NH_4_HCO_3_; 56°C for 45 min) and alkylation (55 mM IAA, 100 mM NH_4_HCO_3_ for 45 min), the samples were precipitated in 80% acetone at −20°C overnight and resuspended in 0.8 M urea and 500 mM TEAB (pH 8.5). Each protein sample (30 μg) was digested with trypsin (protein:trypsin = 30:1) in dissociation buffer (0.1% in TEAB; 37°C for 24 h). The tryptic peptides were labelled with 8-plex iTRAQ reagents (AB Sciex, Foster City, CA, USA) according to the manufacturer’s protocol (iTRAQ113, 114, 115, 116, 117, and 118 for six subgroups of malignant tumours, 119 and 121 for two subgroups of normal controls). The reaction solvents were removed by speed vacuum, and the labelled peptides were dissolved in 20 mM NH_4_FA (pH 10) for further analysis.

### Peptide fractionation by strong cation exchange chromatography and C18 column RP chromatography

High-resolution strong cation exchange chromatography was used to remove the redundant iTRAQ reagents and any interfering substances that might affect MS analysis. Labelled peptides were loaded onto an SCX column (Luna SCX, 4.6 mm × 250 mm, Phenomenex, CA, USA), and eluted by a stepwise linear elution program as follows: 0–10 min equilibration in Buffer A (25% ACN, 20 mM KCl, and 10 mM KH_2_PO_4_, pH 3.0), 10–15 min fast elution with 0–5% Buffer B (25% ACN, 1 M KCl and 10 mM KH_2_PO_4_, pH 3.0), 15–50 min linear elution with 5–30% Buffer B, and 50–55 min washing elution with 30–80% Buffer B. For desalting and further fractionation, the peptide fractions were loaded onto a RP column (Luna C18, 4.6 mm inner diameter × 250 mm length, Phenomenex, CA, USA), and eluted by a step linear elution program as follows: 0–10 min equilibration in 100% solution A [2% acetonitrile (ACN) and 20 mM NH4FA, pH 10], 10–15 min fast elution with 0–12% solution B (80% ACN and 20 mM NH_4_FA, pH 10), 15–50 min linear elution with 12–56% solution B, and 50–55 min washing elution with 56–80% solution B. All of the procedures were performed using a Prominence HPLC system (Shimadzu, Nakagyo-ku, Kyoto, Japan) with a flow rate of 1.0 mL/min and the peptides were monitored at 214 nm. The fractioned peptides were collected at a rate of one tube/min during the linear elution period.

### Peptide analysis by nano-liquid chromatography coupled with Q-exactive mass spectrometry

The peptide fractions were loaded onto a nano RP column (5 μm Hypersil C18, 75 μm × 100 mm, Thermo Fisher Scientific Inc., Waltham, MA, USA) mounted in a Prominence Nano HPLC system (Shimadzu, Nakagyo-ku, Kyoto, Japan). The peptides were eluted with an ACN-gradient from 5–40% containing 0.1% formic acid for 65 min at 400 nL/min. The eluates were transferred to Q-Exactive MS (Thermo Fisher Scientific Inc., Waltham, MA, USA ), which was run in positive ion mode and a data-dependent manner with full MS scan from 350–6000 *m*/*z*, resolution at 70,000, MS/MS scan with minimum signal threshold 17500, and isolation at 2 Da. To evaluate the performance of the mass spectrometry on iTRAQ-labelled samples two MS/MS acquisition modes, higher collision energy dissociation (HCD) and collision induce dissociation (CID), were employed. To optimize the MS/MS acquisition efficiency of HCD, normalized collision energy (NCE) was systemically examined from 25–70%.

### Database search and quantitative data analysis

The raw MS/MS data were converted into MGF format using Proteome Discoverer 1.3 (Thermo Fisher Scientific). The exported MGF files were searched by Mascot 2.3 (Matrix Science, Boston, MA, USA) against the *Uniprot Human 2009–12 database* with a precursor mass tolerance set at 15 ppm and product ion tolerance of 0.02 Da. An automatic decoy database search was performed. Carbamidomethylation of cysteines was set as a fixed modification (C), and oxidation of methionines (M), Gln to pyro-Glu (N-term Q), and 8-plex iTRAQ modifications of N-term, K, and Y were considered variable modifications. A maximum of one miscleavage was accepted.

A protein with at least one unique peptide and a false discovery rate (FDR) <0.01 qualified for further quantification analysis. The fold change in protein abundance was defined as the median ratio of all significantly matched spectra with tag signals. Based on analysis by Proteome Discoverer software, the CV distribution of all quantified proteins and the quantitative results derived from duplicated injections were compared in parallel. The differential expression of all proteins was presented as fold change in iTRAQ ratios. The upregulation of a protein was presented by fold change of at least or more than 1.2 times, and the downregulation by fold change of at least or less than 0.80.

### Bioinformatics analysis by MetaCore™ software

The differentially expressed proteins were further characterized using the software package MetaCore™ 6.18 (http://thomsonreuters.com/metacore, Thomson Reuters) and its online database (https://portal.genego.com) to understand the underlying pathways and protein–protein interaction networks and evaluate the candidate proteins as potential biomarkers.

### Verification analysis by ELISA

The plasma level of candidate proteins was verified on plasma samples from all patients and controls by enzyme-linked immunosorbent assay (ELISA) using commercially available ELISA reagents (USCN Life Science Inc., Wuhan City, China) according to the manufacturer’s instructions. The final data were confirmed by three independent measurements of each plasma protein.

### Statistical analysis

Statistical analysis was performed with SPSS 17.0 for Windows (SPSS Inc, Chicago, IL, USA). Differences were considered statistically significant at p < 0.05. Data derived from the ELISA experiment representing the protein expression levels of different groups were compared using a one-way ANOVA test.

## Results

### Identification of protein expression profile common to tumour patients with Abnormal Savda by Nano-LC coupled with Q-exactive MS

In this study, patients with malignant tumour were classified as either ASt or nASt by diagnosis of Uighur medicine. After the preparation of pooled samples for ASt, nASt, and NC subjects, depletion of high-abundance proteins, enzymatic digestion, and labelling of peptides with iTRAQ reagents, the peptide samples were simultaneously analysed by nanoLC coupled with Q-Exactive MS. This resulted in output of peptide spectra with 95% confidence representing the relative and absolute quantitation of pooled samples.

For analysis of proteomic profiles common to ASt, nASt or NC, we combined the peptide spectra data of pooled samples regardless of tumour type using a functional module of the software Proteome Discoverer. Analysis of peptide spectra setting ≥1.2-fold change as the cutoff for quantitative differences identified 75 and 74 proteins that were differentially expressed in ASt and nASt, respectively, compared with NC. Among them, 34 proteins were upregulated and 26 proteins were downregulated in both ASt and nASt (Additional file 1). This indicates that tumour conditions in both ASt and nASt backgrounds are associated with an aberrantly regulated network of protein expression that is common to different types of tumours. Subsequent quantitative analysis identified a profile of 31 proteins that were differentially expressed between ASt and nASt (Table 1). Analysis of this profile for three cancer types (lung, breast, or gastric cancer) showed that most of these proteins were differentially expressed between ASt and nASt in each cancer type (data not shown). However, among these proteins only 10 were differentially expressed in ASt compared with NC, including upregulation of polymeric immunoglobulin receptor (PIGR), serum amyloid A protein 1 (SAA1), secreted phosphoprotein 2 of 24 kDa (SPP24), orosomucoid 1 (ORM1), lipopolysaccharide-binding protein (LBP), SCAN domain containing 3 (ZNF452/SCAND3), and fatty acid synthase (FASN), and the downregulation of thrombospondin 1 (THBS1) and hyaluronoglucosaminidase 1 (HYAL1) (Table 1). Notably, only three proteins (FASN, LBP and THBS1), all of which were among the proteins differentiating ASt from NC, were differentially expressed in nASt compared with NC.

**Table 1:** **Identification of differentially expressed proteins between ASt and nASt by iTRAQ-based proteomics**

**No**	**Protein information**				**Peptide detection**			**Fold change** ^**a**^		
	**Rec. protein name**	***Uniprot*** **ID**	**Rec. symbol**	**MW**	**Peptide score**	**Peptide coverage**	**Unique peptides**	**ASt/nASt**	**ASt/NC**	**nASt/NC**
1	Polymeric immunoglobulin receptor	P01833	PIGR	83	29.78	1.05	1	3.628	3.002	n.d.
2	Serum amyloid A protein 1	P02735	SAA1	14	409.61	54.1	6	3.201	3.023	n.d.
3	Heat shock 60 kDa protein 1	P10809	HSPD1	61	65.56	3.66	1	2.313	n.d.	n.d.
4	Orosomucoid	P02763	ORM1	24	72.88	18.91	3	1.884	1.233	n.d.
5	SCAN domain containing 3	Q6R2W3	ZNF452	152	13.59	0.38	1	1.629	1.424	n.d.
6	DEAD box polypeptide 41	Q9UJV9	DDX41	70	32.35	0.96	1	1.479	n.d.	n.d.
7	Serpin peptidase inhibitor, antityripsin member 3	P01011	SERPINA3	48	160.91	10.4	4	1.464	n.d.	n.d.
8	Serum amyloid A-4, constitutive	P35542	SAA4	15	103.51	21.54	3	1.452	n.d.	n.d.
9	Fatty acid synthase	P49327	FASN	273	30.66	0.52	1	1.417	2.308	1.675
10	Inter-alpha-trypsin inhibitor heavy chain H3	Q06033	ITIH3	100	139.48	5.84	5	1.364	n.d.	n.d.
11	Alpha-1B-glycoprotein	P04217	A1BG	54	157.16	13.74	6	1.348	n.d.	n.d.
12	Lipopolysaccharide binding protein	P18428	LBP	53	437.6	22.04	9	1.336	1.793	1.345
13	Retinol binding protein 4	P02753	RBP4	23	48.89	4.98	1	1.276	n.d.	n.d.
14	Ribosomal protein S6	P62753	RPS6	29	22.91	6.83	1	1.229	n.d.	n.d.
15	Secreted phosphoprotein 2, 24 kDa	Q13103	SPP24	24	22.13	4.27	1	1.22	1.208	n.d.
16	Fibrinogen gamma chain	P02679	FGG	52	1307.66	63.58	29	1.212	n.d.	n.d.
17	Complement factor H-related protein 1	Q03591	FHR1	38	465.78	36.97	2	1.211	n.d.	n.d.
18	Fibrinogen alpha chain	P02671	FGA	95	2078.11	46.88	40	1.207	n.d.	n.d.
19	Hemopexin	P02790	HPX	52	245.23	22.73	8	1.204	n.d.	n.d.
20	von Willebrand factor	P04275	VWF	309	343.44	4.41	11	0.816	n.d.	n.d.
21	Keratin 2	P35908	KRT2	65	288.46	11.58	6	0.814	0.723	n.d.
22	Pregnancy-zone protein	P20742	PZP	164	422.01	10.86	11	0.803	n.d.	n.d.
23	Apolipoprotein E	P02649	APOE	36	1149.96	66.25	22	0.786	n.d.	n.d.
24	Thrombospondin 1	P07996	THBS1	129	112.25	4.79	4	0.779	0.709	0.825
25	Gelsolin	P06396	GSN	86	225.78	11.64	6	0.762	n.d.	n.d.
26	Insulin-like growth factor-binding protein 3	P17936	IBP3	32	25.36	5.84	1	0.758	n.d.	n.d.
27	Extracellular matrix protein 1	Q16610	ECM1	61	365.78	22.41	9	0.736	n.d.	n.d.
28	Annexin 1	P04083	ANXA1	39	31.01	4.34	1	0.726	n.d.	n.d.
29	Hyaluronoglucosaminidase 1	Q12794	HYAL1	48	40.73	5.52	1	0.657	0.716	n.d.
30	Serpin F1	P36955	PEDF	46	33.05	2.15	1	0.586	n.d.	n.d.
31	Annexin A2 pseudogene 2	A6NMY6	ANXA2P2	39	30.15	4.72	1	0.357	n.d.	n.d.

^a^For a candidate protein, the fold change (ratio) of at least or more than 1.2, and at least or less than 0.80 in plasma content was considered and reported as up- and downregulation, respectively. ASt, Abnormal Savda type tumours; nASt, non-Abnormal Savda type tumours; NC, normal controls; n.d. no difference (fold change of less than 1.2 or more than 0.8); ASt/nASt, fold change of all differentially expressed proteins between ASt and nASt; ASt/NC or nASt/NC, fold change of the same proteins between ASt and NC or nASt and NC as those differentially expressed in ASt/nASt.

### Evaluation of the role and impact of candidate proteins in tumourigenesis by MetaCore™ software and an online database

**Figure 1:**
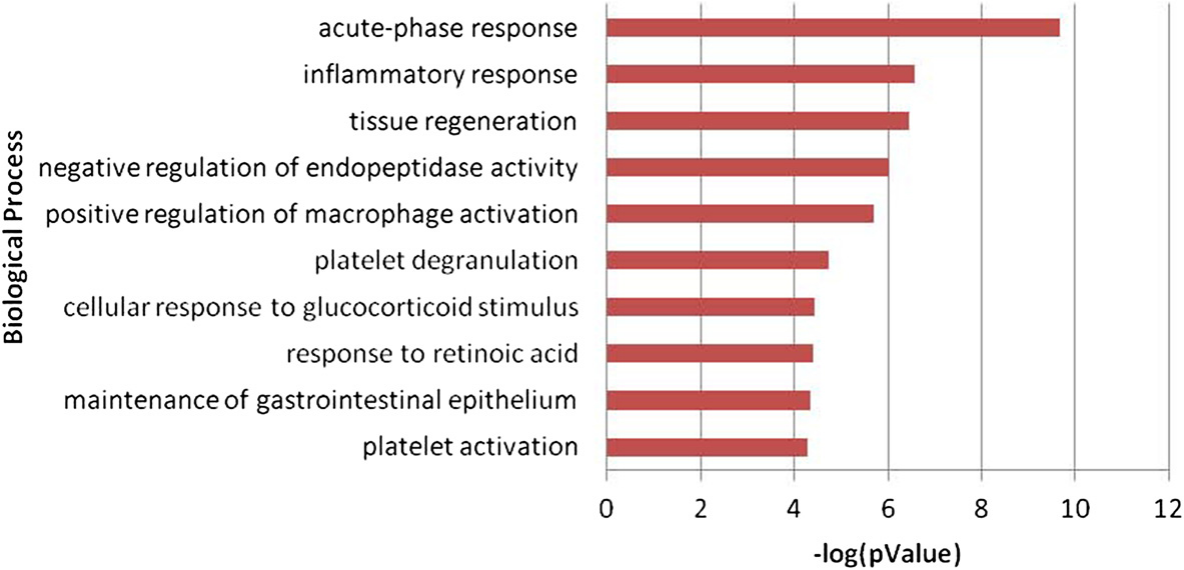
**The role and function of proteins associated with Abnormal Savda type tumours (ASt) in biological processes as displayed by MetaCore™ analysis (** ***p*** **< 0.05).** The analysis includes the function of 31 proteins described in Table 1.

The role of the 31 identified proteins that differentiate ASt from nASt in disease development or tumourigenesis was further evaluated by bioinformatics analysis using MetaCore™ software (version 6.16) and an online database (http://www.genego.com). *Gene Ontology* analysis showed that most of these proteins are localized in the extracellular space, and only small proportions are present at the membrane or in the cytoplasm. These proteins are main players in protein binding or function in the negative regulation of endopeptidase or phospholipase activity, positive regulation of macrophage or platelet activation, and the regulation of acute-phase and inflammatory responses (Figure 1). Some proteins are important regulators of signalling networks or pathways, such as blood coagulation, immune response by multiple TLR signalling, regulation of glucose and lipid metabolism, cell adhesion, transcriptional regulation, or function as effectors in receptor signalling (data not shown). *Biomarker Assessment* analysis based on the *Disease Ontology* database identified most of the proteins as potential biomarkers that had previously been described in other studies. Among them, 17 proteins are involved in stomach disease or neoplasm (P04083, P02649, P49327, P02671, P02679, P06396, P10809, Q12794, P17936, P20742, P02753, P02735, P02735, P35542, P01011, P07996, and P04275) and 22 are potential biomarkers for breast disease and neoplasm, in particular five proteins (P07996, P10809, P17936, Q16610, and Q12794) for breast ductal carcinoma, one (P17936) for hereditary breast cancer, and one (P01011) for fibrocystic breast disease. All proteins except one (A6NMY6) are involved in lung disease or neoplasm, particularly 14 proteins (P01833, P02735, P02649, P02763, P04275, P02679, P06396, P07996, P10809, P17936, P36955, P62753, Q06033, and Q12794) in bronchial neoplasm, three (P17936, P02763, and P62753) in small cell lung carcinoma, and 12 (P02649, P02679, P02735, P04275, P06396, P07996, P10809, Q12794, P17936, P36955, and P62753) in non-small cell lung carcinoma. These findings suggest that the development of Abnormal Savda in tumour patients is probably associated with dysregulation of a whole protein interaction network, and is mainly manifested in aberrant expression of potential biomarkers for malignant tumours.

**Figure 2:**
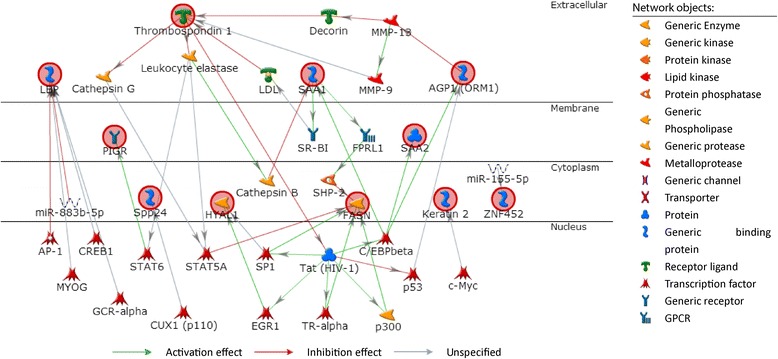
**Network profile for protein interactions associated with 10 candidate proteins in cellular signalling and gene expression by MetaCore™.** The network includes THBS1, LBP, SAA1, ORM1, PIGR, SPP24, HYAL1, FASN, KRT2, and ZNF452 as described in Table 1.

As shown in Table 1, 10 out of the 31 identified proteins may distinguish ASt from nASt and NC and are therefore important candidate biomarkers for ASt. Figure 2 shows the potential interaction and regulation networks associated with these proteins in cellular signalling and gene expression. These proteins are localized in the extracellular space (matrix), membrane, or cytoplasm; are involved in interactions with diverse effectors, such as endopeptidases, matrix metalloproteinases, and phosphatases; and are mainly regulated by transcription factors downstream of distinct signalling pathways.

### Verification of candidate proteins as potential biomarkers for ASt by ELISA

We further verified the results of proteomics analysis by ELISA determination of nine out of the 10 proteins (with the exception of KRT2) that were differentially expressed in ASt compared with both nASt and NC using plasma samples from 136 patients and 40 normal controls. Statistical analysis confirmed significant upregulation of plasma SAA1, SPP24, LBP, and THBS1, and downregulation of PIGR, ZNF452, and FASN in ASt compared with NC (p < 0.05), but no differences were found for ORM1 and HYAL1 (p > 0.05) (Table 2 and Additional file 2). Importantly, the plasma levels of SAA1, PIGR, SPP24, and FASN were also significantly different in ASt compared with nAST (p < 0.05) (Table 2). However, the results of these analyses were not concordant with proteomics data in all cases. For example, expression of PIGR and FASN was upregulated in ASt compared with nASt (or compared with NC) according to iTRAQ proteomics, but ELISA showed significant downregulation of these proteins. In addition, the ELISA analysis showed incremental differences in the expression of some proteins, such as PIGR, ZNF 452, SPP24, and FASN, among the three groups of ASt, nASt, and NC, whereas no difference was found for these proteins when nASt was compared with NC by proteomics. These discrepancies should be clarified by future studies or intensively investigated by consulting other reports in this field.

**Table 2:** **Determination of candidate proteins as potential biomarkers for Abnormal Savda on whole blood (plasma) by ELISA**

	**Plasma protein level (ng/mL)**			**ANOVA test (** ***p*** **value)**			
	**ASt (n = 71)**	**nASt (n = 65)**	**NC (n = 40)**	**F** ***P***	**ASt/nASt**	**ASt/NC**	**nASt/NC**
SAA1	12.755 ± 6.990	9.468 ± 6.965	6.790 ± 4.529	5.645	.035	.002	.150
				.005			
PIGR	37.868 ± 14.336	45.298 ± 14.325	60.734 ± 11.913	13.249	.038	.000	.001
				.000			
ORM1	19.400 ± 5.557	22.217 ± 5.279	20.234 ± 2.940	2.309	.037	.589	.206
				.106			
ZNF452	0.758 ± 0.362	0.701 ± 0.538	1.698 ± 1.112	16.84	.724	.004	.003
				.000			
SPP24	2270.987 ± 304.153	2090.084 ± 264.598	1792.224 ± 266.44	15.057	.018	.000	.001
				.000			
FASN	0.847 ± 0.275	1.129 ± 0.654	2.501 ± 1.114	34.913	.015	.000	.000
				.000			
LBP	204.220 ± 24.523	199.499 ± 46.947	11.609 ± 4.454	146.833	.624	.000	.000
				.000			
HYAL1	12.666 ± 5.069	11.785 ± 5.225	11.351 ± 5.330	4.71	.486	.368	.769
				.626			
THBS1	4.154 ± 2.641	3.366 ± 1.922	1.579 ± 1.100	7.777	.148	.000	.008
				.001			

ASt, nASt or NC, refer to patients with Abnormal Savda type tumours, non-Abnormal Savda type tumours, or normal controls, respectively; ASt/nASt, ASt/NC, nASt/NC, comparative analysis between two groups indicated, with statistical significance at *p* **<** 0.05.

## Discussion

Over time, Uighur medicine has developed relatively complete medical theories, diagnostic methods, and unique prescriptions [2,15]. However, because of a lack of scientific interpretations, Uighur medicine has the same shortcomings as other traditional medicines [32]. The development of proteomics means there is the possibility that serum/plasma can eventually be established as a biomedium for the study of tumour aetiology, drug targets, and personalized cancer care in contemporary medicine [33-35]. In traditional Chinese medicine, many investigators have combined classical concepts with systems biology, in particular applying proteomics to studies of disease aetiology in association with syndromes and multi-target effects of prescriptions [32,36-37]. Thus, it is feasible to study the whole regulation network of protein expression that is altered in the development of different syndromes described in Uighur medicine to reveal the biological basis of these conditions and discover diagnostic biomarkers. This is a holistic concept that is shared by both systems biology and traditional medicine.

Here, we studied the profile of differentially expressed plasma proteins among patients with tumours with Abnormal Savda (ASt), those with tumours and other syndromes (nASt), and healthy individuals as normal controls (NC). Analysis of iTRAQ proteomics data revealed upregulation of 34 proteins and downregulation of 26 proteins in both ASt and nASt compared with NC. Since ASt and nASt are both tumour conditions, these results suggest that ASt and nASt may differ from NC in the shared regulatory networks of protein expression involved in carcinogenesis. Subsequent analysis identified 31 proteins that are differentially expressed in ASt compared with nASt. Most of these proteins are differentially expressed between ASt and nASt in each of three cancer types (lung, breast, or gastric cancer), suggesting that the altered expression of these proteins is associated with Abnormal Savda syndrome. According to bioinformatics analysis, these proteins are mainly localized in the extracellular matrix or cytoplasm, and are involved in many signalling pathways through binding to other proteins or in the inhibition of endopeptidases or phospholipases. Moreover, most of the proteins are known biomarkers for diseases or neoplasms of the stomach, lung, and breast. Thus, the differentially expressed proteins between ASt and nASt are potential biomarkers for malignant tumours associated with Abnormal Savda.

Among these 31 proteins, only 10 proteins showed differential expression that quantitatively distinguished ASt from NC by iTRAQ proteomics. This was partially confirmed by ELISA analysis, which showed that expression of SAA1, SPP24, LBP, THBS1, PIGR, ZNF452, and FASN was significantly altered in the plasma of patients with ASt compared with NC plasma. Importantly, there were significant differences in the expression of SAA1, SPP24, PIGR, and FASN between ASt and nASt and between ASt and NC. Of these proteins, SAA1 is an acute-phase protein that is produced and secreted predominantly by the liver, and associates with high-density lipoprotein (HDL) particles. Plasma SAA1 level is a known biomarker for tumour malignancy, host response, and chemotherapy resistance [38-42]. SAA1 protein expression is upregulated in many common cancers, including lung, ovarian, renal, uterine, and nasopharyngeal cancer [43,44]. High levels of circulating SAA1 are mainly induced by the binding of activated STAT3 and NF-kB transcription factors upon stimulation by inflammation-associated cytokines IL-1 and IL6, and to a lesser extent by the synthesis of this protein in metastatic cells [45-47]. These findings are consistent with the results of our study showing that increased SAA1 expression in patients with malignant tumours is closely associated with Abnormal Savda. Although there were discrepancies in our data for altered expression of PIGR and FASN between iTRAQ and ELISA analysis, other studies have reported the association of both PIGR and FASN expression with tumourigenesis and implied a possible association with Abnormal Savda. PIGR is expressed on mucosal epithelial cells and transports polymeric immunoglobulin A (pIgA) produced by mucosal B cells to the mucosal surface where secretory immunoglobulin A (SIgA) excludes multitudes of dietary, environmental, and microbial antigens to form an initial defence against infection [48]. Consistent with our data from ELISA analysis, downregulation of pIgR is associated with the development of most lung cancers, adenocarcinomas of distal oesophagus and gastro-oesophageal junction, and hepatocellular carcinoma [49-52]. FASN is the key enzyme required for de novo synthesis of fatty acids [53]. The results of iTRAQ proteomics in this study are concordant with previous reports that elevated FASN expression is associated with cancer progression, higher risk of recurrence, shorter survival, and resistance to anticancer therapy of several human cancers, including breast, prostate, kidney, endometrium, colon, lung, and gastric cancer [53-56]. Secreted phosphoprotein 24 kD (SPP24) is a bone matrix protein that is expressed primarily in the liver and bone and binds to and affects the activity of bone morphogenetic proteins (BMPs) [57,58]. SPP24 appears to reduce the growth of tumour cells by binding to both BMP-2 and TGF-β in vitro, but whether plasma SPP24 is upregulated during tumourigenesis in vivo is not currently known [59].

Although the differences in plasma levels of LBP, THBS1, or ZNF452 between ASt and nASt were not confirmed by ELISA, the data from iTRAQ proteomics implicate a role of these proteins to some extent in the development of Abnormal Savda. LBP is an acute-phase protein that initiates an immune response after recognition of bacterial LPS, and mutations in LBP can impair innate immunity [60]. LBP may inhibit TLR2-dependent production of inflammatory cytokines [61]. In conditions of obesity and coronary artery disease LBP expression is targeted by LXRs from macrophages, and serum LBP may serve as an important marker for atherosclerosis. However, the upregulation of circulating LBP in association with tumour malignancy has not been reported previously. THBS1 (TSP1) is a large matricellular glycoprotein that is secreted by many cell types and physically interacts with a variety of ligands during acute and subacute processes [62,63]. The role of THBS1 in tumour growth and metastasis is complicated by its controversial function as a cancer inhibitor or promoter [64]. THBS1 expression is downregulated in advanced non-small cell lung cancer and lymph node metastasis and in breast cancer patients positive for both oestrogen and progesterone receptors [65,66]. In contrast, overexpression of THBS1 in stromal myofibroblasts is associated with tumour growth and nodal metastasis in gastric carcinoma, and may promote tumour cell invasion via upregulation of MMP-9 expression by endothelial cells [67,68]. THBS1 secreted by CD4^+^ and CD8^+^ T cells can negatively regulate angiogenesis in a tumour-bearing mice model [69]. In another study, we demonstrated the upregulation of plasma THBS1 in patients with Abnormal Savda in response to treatment with the prescription for Abnormal Savda, suggesting that downregulation of THBS1 may be associated with Abnormal Savda [70]. There are a limited number of studies on the role of ZNF452 in the plasma and tumour tissue of patients in tumourigenesis, therefore the downregulation of this protein in ASt and nASt compared with controls may provide a novel perspective on the link between cancer progression and Abnormal Savda.

The limitations of this study include verification in a relatively small number of samples from patients (136 cases) and controls (40 cases), the diversity of tumour origin or types, and the restriction of breast cancer to females. Therefore, future validation on a relatively large number of samples is required to establish a standard for the clinical diagnosis of Abnormal Savda among patients with malignant tumours. In addition, independent studies on patients with other tumour types are needed to verify the findings of this study.

## Conclusions

This is the first study of malignant tumours as a complex disease in association with Abnormal Savda at a proteomics level. Our data confirm plasma SAA1, PIGR, SPP24, FASN, and possibly THBS1 as potential biomarkers that are common to different types of malignant tumours and associated with Abnormal Savda. These findings may uncover the mechanisms of tumourigenesis associated with Abnormal Savda, and will contribute to scientific interpretation and standard application of Uighur medicine in the clinical diagnosis and therapy of malignant tumours.

The official terminology ‘abnormal Hilit’ is used in Uighur medicine to describe a syndrome. In this sense, ‘Abnormal Savda’ is interchangeable with ‘Abnormal Savda syndrome’.

### Open access

This article is distributed under the terms of the Creative Commons Attribution License, which permits any use, distribution, and reproduction in any medium, provided the original author(s) and the source are credited.

## Additional files

Additional file 1:
**Identification of plasma proteins that are differentially expressed in ASt and nASt compared with NC by iTRAQ proteomics.**
Additional file 2:
**Determination of candidate proteins as potential biomarkers for Abnormal Savda by ELISA.**

## Abbreviations

iTRAQ: Isobaric tags for relative and absolute quantitation
ASt: Abnormal Savda type tumour
nAST: Non-Abnormal Savda type tumour
NC: Normal controls
SAA1: Serum amyloid A protein 1
SPP24: Secreted phosphoprotein 2 of 24 kDa
LBP: Lipopolysaccharide-binding protein
THBS1: Thrombospondin 1
PIGR: Polymeric immunoglobulin receptor
ZNF452 (SCAN3): SCAN domain containing 3
FASN: Fatty acid synthase
