# Supplementary material for: Identification of plasma protein markers common to patients with malignant tumour and Abnormal Savda in Uighur medicine: a prospective clinical study
Source: BMC Complement Altern Med. 2015 Feb 5;15:9. doi: 10.1186/s12906-015-0526-6 (PMC4321703; doi:10.1186/s12906-015-0526-6)
Supplement: Additional file 1: — Identification of plasma proteins that are differentially expressed in ASt and nASt compared with NC by iTRAQ proteomics. [file 12906_2015_526_MOESM1_ESM.pdf]

Additional file 1- Identification of plasma proteins differentially expressed in ASt and nASt compared with NC by iTRAQ proteomics

No	Protein Information				Peptide Detection			Fold Change [a]	
	rec. Protein name	*Uniprot* ID	rec. Symbol	MW	Peptide Score	Coverage (%)	Unique Peptides	ASt/ NC	nASt/ NC[b]
1	FERM and PDZ domain-containing protein 1	Q5SYB0	FRPD1	173	39.41	0.44	1	3.906	3.319
2	Retinoic acid receptor responder protein 2	Q99969	RARR2	19	48.71	14.11	2	3.105	3.444
3	Putative annexin A2-like protein	A6NMY6	AXA2L	39	30.15	4.72	1	3.092	7.564
4	Serum amyloid A protein	P02735	SAA1	14	409.61	54.10	6	3.023	n.d.
5	Polymeric immunoglobulin receptor	P01833	PIGR	83	29.78	1.05	1	3.002	n.d.
6	Fibulin-5	Q9UBX5	FBLN5	50	133.13	12.50	5	2.312	2.619
7	Fatty acid synthase	P49327	FASN	273	30.66	0.52	1	2.308	1.675
8	Serum amyloid P-component	P02743	SAMP	25	384.02	32.74	8	2.096	2.219
9	Annexin A1	P04083	ANXA1	39	31.01	4.34	1	2.011	2.5
10	Apolipoprotein A	P08519	APOA	501	166.22	16.29	6	1.960	1.294
11	Fibulin-1	P23142	FBLN1	77	851.48	33.57	18	1.887	2.074
12	Lipopolysaccharide-binding protein	P18428	LBP	53	437.6	22.04	9	1.793	1.345
13	Vitronectin	P04004	VTNC	54	506.53	24.27	11	1.772	1.723
14	Histidine-rich glycoprotein	P04196	HRG	60	340.11	24.38	11	1.713	1.678
15	Proteoglycan 4	Q92954	PRG4	151	231.01	5.27	7	1.641	1.601
16	Ficolin-2	Q15485	FCN2	34	224.81	19.17	4	1.606	1.503
17	Extracellular matrix protein 1	Q16610	ECM1	61	365.78	22.41	9	1.574	2.295
18	Apolipoprotein F	Q13790	APOF	33	102.95	8.77	2	1.498	1.72
19	Galectin-3-binding protein	Q08380	LG3BP	65	584.76	27.01	13	1.476	1.697
20	Protein AMB	P02760	AMBP	39	332.87	26.99	8	1.448	1.402

21	Ceruloplasmin	P00450	CERU	122	175.83	8.64	6	1.447	1.931
22	SCAN domain-containing protein 3	Q6R2W3	ZNF452	152	13.59	0.38	1	1.424	n.d.
23	Mannan-binding lectin serine protease 1	P48740	MASP1	79	327.21	19.17	9	1.414	1.486
24	Microfibril-associated glycoprotein 4	P55083	MFAP4	29	33.79	7.06	1	1.399	1.415
25	Ficolin-1	O00602	FCN1	35	62.24	9.20	2	1.390	1.223
26	Insulin-like growth factor II	P01344	IGF2	20	81.36	13.89	2	1.369	1.493
27	Plasma protease C1 inhibitor	P05155	SERPING1	55	352.85	23.20	9	1.355	1.463
28	Inter-alpha-trypsin inhibitor heavy chain H4	Q14624	ITIH4	103	1209.57	35.59	27	1.340	1.475
29	TGFβ-induced protein ig-h3	Q15582	BGH3	75	22.34	1.90	1	1.305	n.d.
30	Vitamin K-dependent protein S	P07225	PROS	75	285.31	15.38	9	1.281	1.225
31	Mannan-binding lectin serine protease 2	O00187	MASP2	76	139.03	4.52	2	1.261	1.308
32	CD5 antigen-like	O43866	CD5L	38	404.58	41.21	11	1.257	n.d.
33	Tenascin	P24821	TENA	241	23.1	0.68	1	1.249	1.256
34	Inter-alpha-trypsin inhibitor heavy chain H2	P19823	ITIH2	106	579.91	17.12	14	1.245	n.d.
35	Orosomucoid	P02763	ORM1	24	72.88	18.91	3	1.233	0.687
36	Pigment epithelium-derived factor	P36955	PED	46	33.05	2.15	1	1.227	2.154
37	Transthyretin	P02766	TTHY	16	334.59	49.66	7	1.221	1.292
38	EGF-containing fibulin-like ECM protein 1	Q12805	FBLN3	55	551.83	32.45	12	1.220	1.376
39	Collectin-11	Q9BWP8	COL11	29	75.73	9.96	3	1.218	1.295
40	Apolipoprotein A-V	Q6Q788	APOA5	41	75.87	5.46	1	1.216	1.293
41	Secreted phosphoprotein 24	Q13103	SPP24	24	22.13	4.27	1	1.208	n.d.
42	Selenoprotein P	P49908	SEPP1	43	166.62	13.91	4	1.204	1.372
43	Coagulation factor V	P12259	FA5	252	225.7	4.18	8	0.831	0.825
44	Sulfhydryl oxidase 1	O00391	QSOX1	83	90.72	6.56	4	0.811	n.d.
45	Apolipoprotein A-IV	P06727	APOA4	45	1781.24	82.32	35	0.809	n.d.
46	Procollagen C-endopeptidase enhancer 1	Q15113	PCOC1	48	81.49	12.92	4	0.806	0.826
47	Apolipoprotein A-II	P02652	APOA2	11	410.37	69.00	9	0.790	0.804
48	Apolipoprotein A-I	P02647	APOA1	31	1761.85	83.15	36	0.779	n.d.
49	Apolipoprotein C-I	P02654	APOC1	9	214.97	37.35	6	0.772	n.d.

50	Adiponectin	Q15848	ADIPOQ	26	24.99	6.15	1	0.761	0.67
51	Apolipoprotein C-II	P02655	APOC2	11	310.21	52.48	5	0.742	n.d.
52	Retinol-binding protein 4	P02753	RET4	23	48.89	4.98	1	0.736	0.593
53	Keratin, type II cytoskeletal 2 epidermal	P35908	KRT2	65	288.46	11.58	6	0.723	n.d.
54	Inter-alpha-trypsin inhibitor heavy chain H1	P19827	ITIH1	101	126.36	6.37	5	0.722	0.782
55	Apolipoprotein C-III	P02656	APOC3	11	257.19	55.56	5	0.718	0.827
56	Hyaluronidase-1	Q12794	HYAL1	48	40.73	5.52	1	0.716	n.d.
57	Thrombospondin-1	P07996	THBS1	129	112.25	4.79	4	0.709	0.825
58	Keratin, type I cytoskeletal 10	P13645	KRT10	59	441.1	25.34	11	0.698	0.787
59	Apolipoprotein C-IV	P55056	APOC4	15	105.82	32.28	4	0.672	0.745
60	Coagulation factor XIII A chain	P00488	F13A	83	89.07	4.51	2	0.657	0.729
61	Glutathione peroxidase 3	P22352	GPX3	26	183.4	31.42	6	0.649	0.697
62	Keratin, type II cytoskeletal 1	P04264	KRT1	66	373.28	16.61	9	0.632	0.592
63	Keratin, type I cytoskeletal 9	P35527	KRT9	62	76.22	4.01	2	0.621	0.636
64	Alpha-2-antiplasmin	P08697	SERPINF2	55	75.58	6.31	3	0.616	0.7
65	Probable ATP-dependent RNA helicase DDX41	Q9UJV9	DDX41	70	32.35	0.96	1	0.597	0.415
66	Tetranectin	P05452	TETN	23	45.34	5.94	1	0.592	0.615
67	Fibrinogen gamma chain	P02679	FGG	52	1307.66	63.58	29	0.535	0.46
68	Inhibin beta C chain	P55103	INHBC	38	28.44	2.27	1	0.534	0.599
69	Fibrinogen alpha chain	P02671	FGA	95	2078.11	46.88	40	0.532	0.458
70	Platelet factor 4	P02776	PLF4	11	72.64	25.74	2	0.526	0.581
71	Fibrinogen beta chain	P02675	FGB	56	1685.1	66.19	32	0.508	0.453
72	Transcription factor IIIB 90 kDa subunit	Q92994	TF3B	74	33.69	1.18	1	0.330	0.341
73	Coagulation factor XIII B chain	P05160	F13B	76	162.39	12.25	5	0.312	0.352
74	Prothrombin	P00734	THRB	70	526.43	28.14	15	0.149	0.19
75	Cadherin-5	P33151	CDH5	88	28.21	1.40	1	0.101	0.097

[a] For a candidate protein, the fold change (ratio) of at least or more than 1.2, and at least or less than 0.8 in plasma content was considered and reported as up- and downregulation, respectively. ASt, Abnormal Savda type tumours; nASt, non-Abnormal Savda type tumours; NC, normal controls; n.d.no difference; ASt/NC, fold change of all differentially expressed proteins between ASt and NC. nASt/NC, fold change of the same proteins between nASt and NC as those differentially expressed in ASt/NC.
