# Supplementary material for: Identification of plasma protein markers common to patients with malignant tumour and Abnormal Savda in Uighur medicine: a prospective clinical study
Source: BMC Complement Altern Med. 2015 Feb 5;15:9. doi: 10.1186/s12906-015-0526-6 (PMC4321703; doi:10.1186/s12906-015-0526-6)
Supplement: Additional file 2: — Determination of candidate proteins as potential biomarkers for Abnormal Savda by ELISA. [file 12906_2015_526_MOESM2_ESM.pdf]

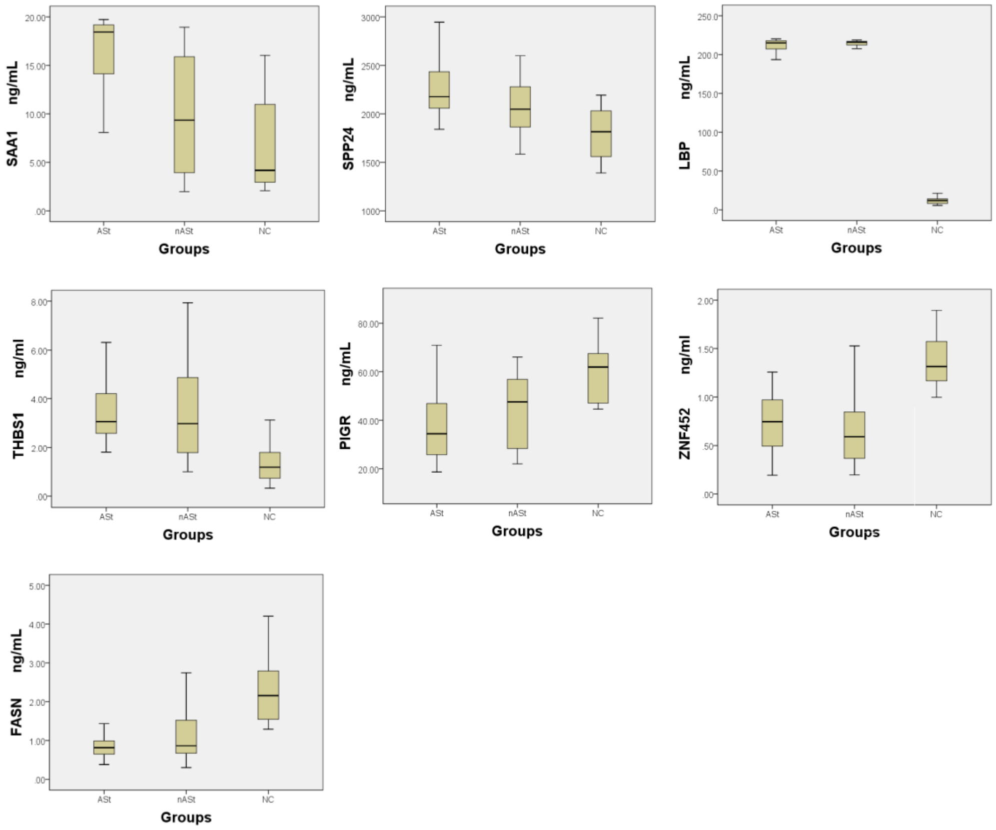

Additional file 2- Determination of candidate proteins as potential biomarkers for Abnormal Savda by ELISA. ASt, nASt and NC, represents the result of ELISA determination and statistical analysis (significance at $p<0.05$) as described in Table 2. ASt, Abnormal Savda type tumours; nASt, non-Abnormal Savda type tumours; NC, normal controls.
